# Ongoing barriers to immediate postpartum long-acting reversible contraception: a physician survey

**DOI:** 10.1186/s40834-018-0078-5

**Published:** 2018-11-08

**Authors:** Emily C. Holden, Erica Lai, Sara S. Morelli, Donald Alderson, Jay Schulkin, Neko M. Castleberry, Peter G. McGovern

**Affiliations:** 1 Obstetrics, Gynecology and Women’s Health, Rutgers-New Jersey Medical School, 185 South Orange Avenue, E-level, Newark, NJ 07103 USA; 2 Reproductive Endocrinology and Infertility, University Reproductive Associates, 214 Terrace Avenue, Hasbrouck Heights, NJ 07604 USA; 3 Rutgers University Biostatistics and Epidemiology Services Center, Rutgers University, 65 Bergen St, Newark, NJ 07103 USA; 4 Department of Obstetrics and Gynecology, University of Washington, Box 356460, Seattle, WA 98195-6460 USA; 5 American College of Obstetricians and Gynecologists, 409 12th Street, SW, Washington, DC 2002420024-9998 USA

**Keywords:** Immediate postpartum long-acting reversible contraception

## Abstract

**Background:**

Postpartum women are at risk for unintended pregnancy. Access to immediate long-acting reversible contraception (LARC) may help decrease this risk, but it is unclear how many providers in the United States routinely offer this to their patients and what obstacles they face. Our primary objective was to determine the proportion of United States obstetric providers that offer immediate postpartum LARC to their obstetric patients.

**Methods:**

We surveyed practicing Fellows and Junior Fellows of the American College of Obstetricians and Gynecologists (ACOG) about their use of immediate postpartum LARC. These members are demographically representative of ACOG members as a whole and represent all of the ACOG districts. Half of these Fellows were also part of the Collaborative Ambulatory Research Network (CARN), a group of ACOG members who voluntarily participate in research. We asked about their experience with and barriers to immediate placement of intrauterine devices and contraceptive implants after delivery.

**Results:**

There were a total of 108 out of 600 responses (18%). Participants practiced in a total of 36 states and/or US territories and their median age was 52 years. Only 26.9% of providers surveyed offered their patients immediate postpartum LARC, and of these providers, 60.7% work in a university-based practice. There was a statistically significant association between offering immediate postpartum LARC and practice type, with the majority of providers working at a university-based practice (*p* < 0.001). Multiple obstacles were identified, including cost or reimbursement, device availability, and provider training on device placement in the immediate postpartum period.

**Conclusion:**

The majority of obstetricians surveyed do not offer immediate postpartum long-acting reversible contraception to patients in the United States. This is secondary to multiple obstacles faced by providers.

## Background

In recent years in the United States, there has been a decline in the unintended pregnancy rate from 51 to 45% [1]. Although this change represents an improvement, the United States continues to have higher rates of unintended pregnancy than many parts of northern and Western Europe [2]. As a result, improving women’s health by decreasing unintended pregnancies remains one of the goals of the Centers for Disease Control and Prevention [3]. Increasing patient access to long-acting reversible contraception (LARC) is one method which may aid in reducing the unintended pregnancy rate further. Specifically, immediate postpartum LARC placement, or LARC placement prior to hospital discharge, may help decrease the unintended pregnancy rate.

Unfortunately, only about a third of women who desire postpartum LARC will ultimately obtain it by 8–12 weeks postpartum, if they do not obtain it before hospital discharge [4–6]. This has been demonstrated with regards to both intrauterine devices and contraceptive implants. Not only are these women at risk for unintended pregnancy but they are also at risk for short inter-pregnancy interval, even if they are given an alternative contraceptive in the interim [4].

Given the potential benefits of immediate postpartum LARC, ACOG recommends counseling women prenatally about the options of immediate postpartum LARC and offering immediate postpartum LARC as an effective option for postpartum contraception [7]. Despite these recommendations, it remains unclear what proportion of U.S. obstetricians offer immediate postpartum LARC to their patients. We hypothesize that despite ACOG’s recommendations regarding immediate postpartum LARC, many providers are not offering it to their patients.

## Methods

We conducted an online survey to assess whether physicians who provide obstetric care in the United States were offering immediate postpartum LARC to their patients. This included any commercially available intrauterine device or subcutaneously placed contraceptive implant. The survey was distributed to 600 physicians who were practicing Fellows and Junior Fellows of the American College of Obstetricians and Gynecologists. Of these 600 physicians surveyed, 300 were randomly selected members of the Collaborative Ambulatory Research Network (CARN), a group of 1400 ACOG members who voluntarily participate in research. These members are demographically representative of ACOG members as a whole and represent all of the ACOG districts [8]. 300 additional surveys were sent to randomly selected ACOG Fellows who are not part of CARN.

The initial study e-mail was sent in May 2017, and data collection ended July 2017. Providers received up to six reminder e-mails. Emails included a link to opt-out of participation. Respondents who did not provide obstetric care were ineligible to participate and, therefore, excluded.

The primary study outcome assessed was whether obstetric providers are offering immediate postpartum LARC to patients. The secondary study outcome assessed was identification of obstacles which providers face with regards to offering immediate postpartum LARC. These included obstacles surrounding adequate training, reimbursement, LARC device availability, and concern about intrauterine device expulsion. Demographic criteria were collected for all participating providers.

Statistical analysis was performed using both SAS 9.4, SAS Institute Inc., Cary, NC, and Microsoft Excel, Version 14.7.6. *P*-values are the result of Fisher’s exact test for categorical variables, Wilcoxon-Mann-Whitney test for continuous variables. A *p*-value of less than 0.05 was considered significant. This study was approved by the Rutgers Health Sciences Institutional Review Board (Newark, NJ).

## Results

**Fig. 1:**
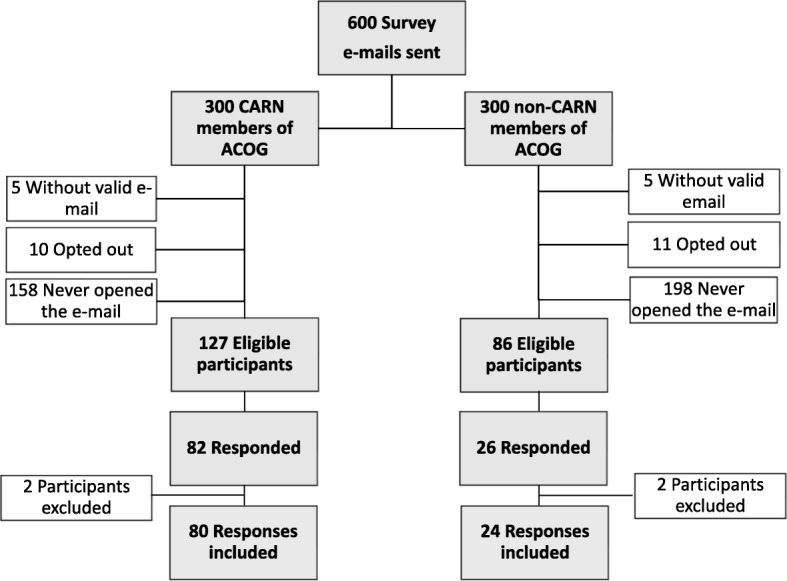
Participants included in the study. A total of 4 participants who completed the survey were excluded because they did not meet inclusion criteria. *CARN* Collaborative Ambulatory Research Network. *ACOG* American College of Obstetrics and Gynecology

Of the 600 survey e-mails sent out, there were 108 responses (18%) (Fig. 1). Four of the participants did not provide obstetric care and were excluded from the study. Eighty-two of the 104 participants, or 79%, were CARN members. Demographic data is shown in Table 1. The mean age of survey respondents was 52 years old, with a range from 32 to 76 years old. Respondents were based in 36 states and/or US territories.

**Table 1:** Demographic characteristics of survey participants

	All (*N* = 104)	Provides IP LARC (*N* = 28)	Does not provide IP LARC (*N* = 76)	*p* - values
Median Age (Years)	52	55	49	*p* = 0.21
Median Years in Practice	19.5	23	15.5	*P* = 0.30
Racial/ethnic group				
White	81.7%	82.1%	81.6%	*P* = 0.61
Black or African American	4.8%	3.6%	5.3%	
Hispanic or Latino	1.0%	3.6%	0	
Asian	9.6%	7.1%	10.5%	
American Indian or Alaskan Native	0	0	0	
Native Hawaiian or Other Pacific Islander	0	0	0	
Multiracial	2.9%	3.6%	2.6%	
Practice Location				
Urban, Inner City	23.1%	50.0%	13.2%	*p < 0.001
Urban, Non-inner City	23.1%	32.1%	19.7%	
Suburban	34.6%	10.7%	43.4%	
Mid-sized	11.5%	3.6%	14.4%	
Rural	7.7%	3.6%	9.2%	
Practice Setting				
Solo private practice	6.7%	0	9.2%	*p < 0.001
OB-GYN Partnership Group	36.5%	7.1%	47.4%	
Multispecialty Group	11.5%	10.7%	11.8%	
Military/Government	1.9%	7.1%	0	
University Based	26.9%	60.7%	14.4%	
HMO Staff Model	5.8%	3.6%	6.6%	
Other	10.8%	10.7%	10.5%	

*P*-values are the result of Fisher’s exact test for categorical variables, Wilcoxon-Mann-Whitney test for age and years in practice. *p-values < 0.05 are considered significant

Of the 104 respondents, 97 (93.3%) placed intrauterine devices (IUDs) and 88 (84.6%) placed etonogesterel contraceptive implants in their practice. However, only 28 (26.9%) providers surveyed provide immediate postpartum LARC to their obstetric patients. On the other hand, 84 (80.8%) providers in the study offered placement at the time of their first postpartum visit.

Of the 76 providers who did not offer immediate postpartum LARC, most reported multiple barriers including lack of IUD device availability, lack of implant device availability, problems with cost or reimbursement, a lack of training to place immediately postpartum IUDs, and concern over high expulsion rates of IUDs (Table 2). Interestingly, 11 (14.5%) participants who do not offer immediate postpartum LARC reported that their patients are not interested in this method. Furthermore, 59 (77.6%) providers who did not currently offer immediate postpartum LARC would either like to offer or would consider offering this method of contraception in the future. Similarly, 65 (85.5%) of these providers would either like to participate or would consider participating in training to place immediate postpartum IUDs.

**Table 2:** Physician’s perceived barriers to offering immediate postpartum LARC amongst providers who DO NOT offer it

Perceived Barriers to Immediate Postpartum LARC	Response Rate *N* = 76
Implant Device availability	55 (72.4%)
IUD Device Availability	52 (68.4%)
Cost or Reimbursement of IUDs	41 (53.9%)
Cost or Reimbursement of implants	44 (57.9%)
Lack of training to place IP-IUDs	36 (47.4%)
High expulsion rate of IUDs	29 (38.2%)
Lack of patient interest	11 (14.5%)
Other*	14 (18.4%)

Participants could select multiple barriers. *Other common barriers reported include a high follow-up of postpartum patients negating the necessity of immediate postpartum placement (*N* = 3) and working in a Catholic hospital (*N* = 3). *LARC* Long acting reversible contraception, *IUD* intrauterine device, *IP* immediate postpartum

Of the 28 providers who do offer immediate postpartum LARC, survey respondents reported similar barriers including cost or reimbursement (57.1%), availability of devices (42.9%), and lack of patient interest (14.3%). There was no significant relationship between offering immediate postpartum LARC and provider age. Of the providers who did offer immediate postpartum LARC to their patients, 17 (60.7%) worked in a university-based practice. There was a significant relationship between offering immediate postpartum LARC and practice type with the majority of providers working at a university-based practice (*p* < 0.001). Providers working at a university-based practice were significantly more likely to offer immediate postpartum LARC than providers working in any other practice setting (*P* < 0.001).

The survey also assessed participants’ knowledge about immediate postpartum LARC placement. When the participants were asked the optimal time frame to place an immediate postpartum IUD, only 56 (53.8%) providers gave the correct response of placing one within ten minutes of delivery of the placenta.

## Discussion

Despite the recommendation by ACOG to offer immediate postpartum LARC to obstetric patients, most of our survey participants do not. University-based physicians were significantly more likely to offer immediate postpartum LARC to their patients than those based in other practice settings. On the other hand, there was no association between offering immediate postpartum LARC and age of the provider or number of years in practice.

Compared to a 2014 physician survey of ACOG members where only 7% of obstetricians provided immediate postpartum IUD placement, our survey indicates a modest increase in obstetricians providing immediate postpartum LARC [9]. However, the overall percentage of obstetricians providing this service remains low. This finding remains consistent across other obstetric providers, including midwives and family medicine physicians [10, 11] and continues to highlight the need for increased training related to immediate postpartum LARC.

This survey identifies multiple perceived barriers which providers face when considering offering immediate postpartum LARC. The most common barrier we identified was lack of access to devices on labor and delivery and on the postpartum unit. Other barriers include cost or reimbursement and a lack of training of physicians in placement of immediate postpartum LARC. Despite these barriers, most of the providers still desired training and the ability to offer their patients immediate postpartum LARC.

A strength of our study is that our sample includes physicians from across the country with a wide age range and variety of practice situations. Although our sample size is small, the majority of respondents were involved with CARN, a research network, and therefore were more likely to be affiliated with a University practice. Thus, it is likely that any selection bias present would be biased towards providers being more likely to offer immediate postpartum LARC. Additionally, although it is important to understand the role of perceived barriers in contributing to the physician’s decision to provide immediate postpartum LARC, more studies are needed to better understand how such barriers affect implementation of this service.

Many women in the postpartum period resume sexual intercourse before 6 weeks [12–15] with one study reporting 15.2% of women engaging in intercourse within four weeks after delivery [15]. Reported incidences of resumption of sexual intercourse range from 27.6 to 62% by 6 weeks [14–16]. Given that women may start ovulating as early as 25 days postpartum, this places them at increased risk for unintended pregnancy if they are not using reliable contraception. Furthermore, since many women are not seen for their postpartum visit until after possible ovulation, they are unlikely to have access to reliable contraception between hospital discharge and their postpartum visit. Harney et al. found that 38% of women who had planned for postpartum LARC did not attend their postpartum visit, with 11.4% conceiving after a short inter-pregnancy interval [4]. It is well established that a short inter-pregnancy interval is associated with increased maternal and fetal complications including increased rates of preterm delivery and preeclampsia [17–19]. Increasing access to LARC in the immediate postpartum period decreases a woman’s chance of short inter-pregnancy interval during the postpartum period [20]. In addition to decreasing unintended pregnancies, there are potential cost savings which may result from increased access to LARC [21, 22].

To begin to address the existing barriers, some research has been performed on implementation of successful immediate postpartum LARC programs. Hofler et al. suggest that successful strategies for implementation of immediate postpartum LARC include a multidisciplinary approach with three essential elements: early involvement of multidisciplinary team members consisting of those who provide direct clinical care, pharmacy and billing personnel; early reassurance and understanding of hospital financial status; and ongoing effective team communication with establishment of clear roles and responsibilities [23]. However, due to the many complexities of theses systems and variations between states, programs will likely need to trouble shoot the obstacles which arise [23].

## Conclusion

The majority of obstetricians surveyed do not offer immediate postpartum LARC to patients in the United States. This occurs despite the recommendations of ACOG to offer it to all obstetric patients, and seems to be secondary to multiple obstacles that providers face. We must continue to seek ways to overcome these obstacles, in order to improve implementation of this vital family planning option.

## Abbreviations

ACOG: American College of Obstetricians and Gynecologists
CARN: Collaborative Ambulatory Research Network, a subset of the American College of Obstetricians and Gynecologists
IUD: Intrauterine device
LARC: Long-acting reversible contraception
